# The Development of Processing Methods and Materials Used for Non-Pneumatic Tires: A Review

**DOI:** 10.3390/ma17225660

**Published:** 2024-11-20

**Authors:** Meng Sun, Haolong Zhong, Kangpei Qin, Ting Xu, Wengang Yang, Yu Zhang, Lei Lu

**Affiliations:** 1 Ji Hua Laboratory, Foshan 528200, China; sunm@jihualab.ac.cn (M.S.); zhonghaolong@jihualab.ac.cn (H.Z.); qinkp@jihualab.ac.cn (K.Q.); 2 Changchun Institute of Applied Chemistry, Chinese Academy of Sciences, Changchun 130024, China; zhyu@ciac.ac.cn; 3 China FAW Co., Ltd., Changchun 130011, China; lulei8@faw.com.cn

**Keywords:** non-pneumatic tire, classification, materials research, manufacturing techniques, future development

## Abstract

Non-pneumatic tires (NPTs) have garnered significant attention due to their advantages, such as energy efficiency, safety, versatile applications, and superior performance, compared to traditional rubber-based pneumatic tires (PTs). This mini review provides a concise overview of NPTs, beginning with their definition, structural design, and classification based on structural variations. The review then examines recent advancements in the materials used for NPTs, including those for the tread, elastic support structure, skeleton, and adhesives, with a focus on their specific properties. Furthermore, it summarizes various manufacturing techniques such as compression molding, centrifugal casting, injection molding, 3D printing, and mechanical assembly. Lastly, the review outlines the general manufacturing procedures of NPTs, discusses the challenges currently faced by the technology, and offers insights into future development directions. This mini review aims to support NPT researchers and practitioners, particularly in the fields of process and materials engineering, in advancing their work on NPTs.

## 1. Introduction

Tires constitute a pivotal automotive component that serves as the essential interface between vehicles and the road surface. They fulfill a diverse spectrum of critical functions, encompassing the transmission of driving forces to the road, bearing vehicular loads, ensuring precise directional control along the intended trajectory, and providing comfort to passengers, among others. The configuration of tires has continuously evolved in tandem with technological advancements. At present, pneumatic tires (PTs) reign supreme within the industry and have experienced widespread use for over a century. This longevity can be attributed to their commendable attributes, such as exceptional elasticity, as well as their proficiency in shock absorption and cushioning. Nevertheless, the inherent advantages of PTs are offset by certain drawbacks, including intricate manufacturing processes, elevated production costs, susceptibility to punctures and blowouts, and unsuitability for deployment in extremely harsh environmental conditions. These limitations not only impede the broader adoption of PTs but also curtail prospects for enhancing production efficiency [1]. Compelling data underscore the gravity of the situation, with approximately 70% of highway traffic accidents being attributed to tire blowouts. Remarkably, at speeds exceeding 160 km/h, nearly 100% of traffic accidents resulting in fatalities are directly attributable to tire blowouts [2]. In light of safety imperatives, the concept of non-pneumatic tires (NPTs) has emerged as a prospective alternative.

In the nascent stages of its evolution, NPTs were often simplistically regarded as solid tires. However, as technological advancements unfolded and cognitive perspectives shifted, the scope of NPTs has significantly broadened, now encompassing tires with hollow structures. Thanks to the replacement of air with elastic supports or entities, some types of NPTs tend to yield a slew of advantages, including the elimination of run-flat risks, obviating the need for air maintenance, ameliorating rolling resistance, and enhancing passenger comfort [3]. As a result, vehicles equipped with NPTs obviate the necessity of carrying a spare tire, thereby reducing the overall vehicle weight and ameliorating fuel efficiency [4]. The most significant benefit of this design potentially lies in its capacity to broaden the spectrum of feasible design options. That expansion includes the elimination of some of the most restrictive constraints imposed by pneumatic tire mechanics, including the coupling of stiffnesses, restrictions on size and shape, and containment of the large potential energy associated with pressurized inflation gas [5].

**Figure 1:**
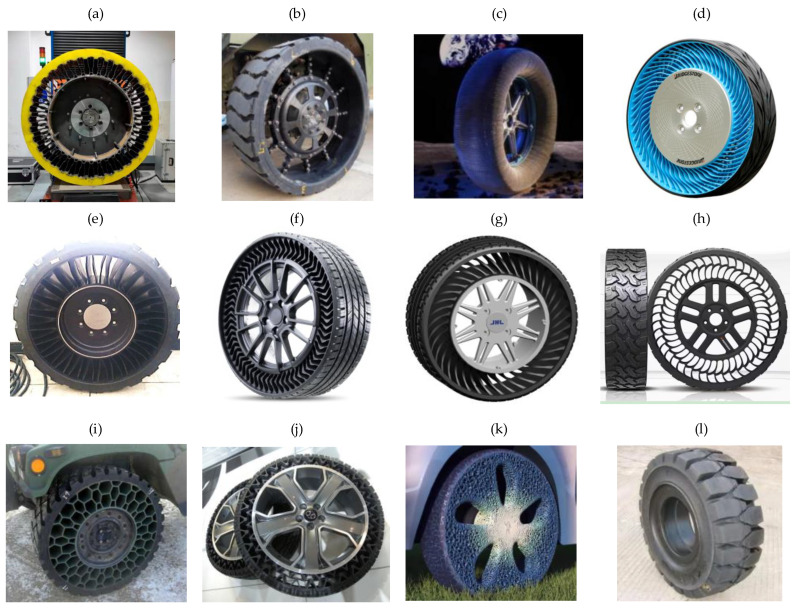
Representative examples of NPTs: (**a**) a high-load-capacity NPT [10]; (**b**) a mechanical elastic support type NPT [11]; (**c**) NASA’s Superelastic Tire made of a shape memory alloy [11]; (**d**) an Air Free Concept Tire [8]; (**e**) a Michelin Tweel [12]; (**f**) a Michelin UPTIS [7]; (**g**) a passenger car NPT designed by Jihua Laboratory; (**h**) i-Flex [9]; (**i**) honeycomb [13]; (**j**) an NPT with a negative Poisson’s ratio [11]; (**k**) a 3D-printed NPT [14]; (**l**) a solid-type NPT.

In 2005, Michelin^®^ introduced the pioneering commercialized NPT, named Tweel, which is a landmark development that signified the pivotal transition of NPT concepts from the realm of theoretical design to the realm of tangible physical production [6]. Michelin also cooperated with General Motors Corporation, jointly unveiling the Uptis NPT, and created plans to incorporate it into passenger vehicles by 2024 [7]. Another prominent tire manufacturer, Bridgestone^®^, introduced Air Free Concept Tires in both their first and second generations in 2012 and 2014, respectively. These innovations were accompanied by assertions of heightened load-bearing capabilities, enhanced driving performance, and an overarching commitment to environmental considerations, as exemplified by eco-design principles [8]. Hankook^®^ also embarked on the pursuit of NPT technology as early as 2010, with continuous research and development efforts dedicated to refinement. In 2022, Hankook unveiled the “i-Flex” NPT at Hyundai Rotem’s exhibition booth in the Defense & Security Expo Korea (DX KOREA). This NPT variant boasts a dual-arch structure 17-inches in size and specifically designed for application in multi-purpose unmanned ground vehicles (UGVs) [9]. Representative NPT products are visually delineated in Figure 1 for reference.

Considerable attention has recently been directed toward the array of advantages associated with NPTs. Nonetheless, the reason for the limited widespread adoption of NPTs can be attributed to their enduring deficiencies. In direct comparison to PTs, NPTs exhibit certain disadvantages, including an elevated weight, challenges in maintaining balance at higher speeds, susceptibility to bonding failures, and, notably, higher cost implications [15]. Furthermore, within the domain of NPTs, ongoing discourse among researchers and engineers persists regarding various issues, encompassing the modeling of vibrations at high speeds and the establishment of reliable methodologies for the prediction of NPT failure mechanisms [16,17]. Nevertheless, it is imperative to underscore that NPTs remain a compelling choice in industries such as agriculture, mining, and construction, where the imperative of minimizing tire maintenance downtime serves as a catalyst for bolstering productivity efficiency and harnessing the full spectrum of NPT advantages [18].

While an increasing number of researchers and companies worldwide have shown a growing interest in non-pneumatic tires (NPTs), the prevailing focus has predominantly centered on the intricacies of structural design and mechanical behavior simulations. In fact, it is essential to underscore that the diverse structural designs and mechanical attributes of NPTs are intricately intertwined with the judicious selection of processing methodologies and the materials employed in their fabrication. Consequently, the choice of processing methods and materials represents a foundational and indispensable element in the manufacture procedure of NPTs, particularly when considering industrial-scale production. This paper serves to offer a concise introduction to the concept and classification of NPTs while providing a summative overview of the evolving landscape of processing methods and materials essential to NPT development. In order to enable a comprehensive understanding for R&D practitioners and researchers, the contents of this paper draw substantially from research findings, scientific reports, and pertinent internet-based reports. In conclusion, this paper not only presents a summation of the current state and challenges within the NPT industry but also offers forward-looking insights into the future trajectory in this field.

## 2. Concept and Classification of NPTs

### 2.1. Concept

At present, a precise and universally accepted definition for NPTs remains elusive, beyond the overarching notion that NPTs are tires designed to support vehicles without reliance on air inflation. Furthermore, a variety of commercial nomenclature has emerged, including designations such as airless tires, air-free tires, non-inflatable tires, and solid tires, among others. In strict academic parlance, NPTs represent a category of wheel systems that employ elastic support structures to supplant the pneumatic inflation characteristic of PTs. Consequently, there exists an ongoing scholarly debate concerning whether solid tires should be encompassed within the purview of NPTs. This paper, in pursuit of a comprehensive understanding, adopts an inclusive perspective with regard to NPTs, and henceforth, all the aforementioned nomenclature are uniformly called NPTs.

### 2.2. Composition Structure

**Figure 2:**
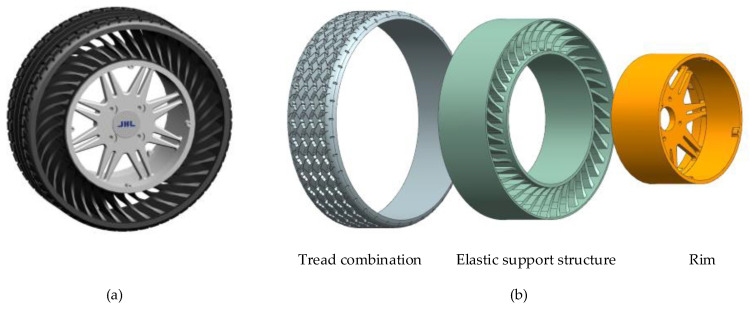
NPT: (**a**) physical sample of NPT; (**b**) composition structures of NPTs.

To enhance the study of the mechanical properties and optimize the structural design of non-pneumatic tires, researchers typically decompose the tire structure into the following components for detailed analysis: tread, shear band, support structure, and rim [19,20,21]. The tread is the part of the tire that comes into direct contact with the ground. Its primary function is to provide grip and traction while resisting wear and tear. The shear belt is also called the reinforcing belt layer. Its main function is to tighten the non-pneumatic tire and ensure the overall circumferential stiffness of the non-pneumatic tire. It is embedded in the tread, as shown in Figure 2, so in this article, it is referred to as the tread combination together with the tread. The support structure is the core part of the non-pneumatic tire. It is responsible for bearing the weight of the vehicle and the impact of the road, providing the necessary support and elasticity, which is the role of the tire pressure of the pneumatic tire [1,4]. When designing the support structure, it is necessary to find a balance between stiffness and flexibility to ensure that it can perform well under different loads and road conditions [22]. The rims of NPTs serve the critical function of affixing to the vehicle’s hub, thereby facilitating the transmission of driving forces from the vehicle to the tires. It is noteworthy that, in contrast to the assembly of pneumatic tires, the rims of NPTs constitute an integral part of the NPT structure, establishing connections with other tire components through either bonding or mechanical fixation.

### 2.3. Classification

The presence of an elastic support is a significant distinctive factor between NPTs and PTs. The elastic support structure consists of various elastic units. This component determines the mechanical properties of NPTs. Unlike the isotropic tire pressure that provides support in PTs, the elastic support structure provides a variety of design possibilities for NPTs and even decouples mechanical properties.

NPTs can be classified based on their load-bearing methods into bottom load-bearing, top load-bearing, and global load-bearing types. According to application scenarios, they can be divided into military NPTs, NPTs for engineering and construction vehicles, and agricultural machinery NPTs, among others. Based on the materials used, NPTs can be categorized into rubber NPTs, polyurethane NPTs, and composite NPTs. This paper classifies NPTs based on their structural characteristics. NPTs designed based on elastic support structures are mainly divided into three categories: the mechanical elastic support type, elastomer elastic support type, and solid type.

Mechanical elastic support-type NPTs, as the nomenclature implies, rely on elastic support structures or hinge units constructed from metallic materials to provide support [10,23,24,25]. This type of NPT has a strong load-bearing capacity and is suitable for areas with medium/low speed and medium/high load.

Elastomer elastic support non-pneumatic tires are the most frequently studied and the most common type, with a variety of design types, including but not limited to the spoke type, honeycomb type, cross-support type, etc. Spoke-type NPTs feature multiple elastic line or flexuous spokes that are uniformly distributed along the circumference of tires. Typically situated between the inner and outer buffer layers of the elastic support, these spokes undergo elastic deformation, providing critical support and absorbing impact forces when the tire is in motion on the road [26,27]. Honeycomb-type NPTs represent a specific category of non-pneumatic, explosion-proof safety tires, initially developed by the United States-based Cooper company, and they are designed into hexagonal and circular honeycomb structures based on the results of the mechanical properties simulation [28,29,30]. Cellular-type NPTs, also referred to as hexagonal spoke-type or lattice-based NPTs, incorporate a distinctive support structure, comprising cells with solid edges or faces, often arranged in a regular or variable pattern [31,32]. This structure affords enhanced circumferential deformations, thereby diminishing the circumferential stiffness [33]. Two or more spokes cross at a designed angle to form a cross-supported NPT, which also accounts for a large proportion [34,35,36]. In addition to the above types, there are many other structural types of NPTs, which is also a manifestation of the diversification of non-pneumatic tires in design [37,38]. Elastomer elastic support NPTs are the most common and most frequently studied because they have a variety of designs and rich elastomer materials. Compared with the other two NPTs, they are more suitable for areas with medium speed and load or areas with high speed and light load.

Solid-type NPTs, representing the most elementary structural variant within the NPT field, are primarily composed of solid rubbers or other elastomers serving as the elastic supports [39,40,41]. In some instances, microcellular foamed rubbers or alternative elastomers are employed to reduce the overall tire mass [42]. Solid tires can provide excellent durability, puncture resistance, and low maintenance, thereby maintaining efficient performance under harsh operating conditions. They are very suitable for areas with low speed but high load, such as forklifts, pallet trucks, lifts, and other industrial and warehousing equipment.

According to this classification of NPTs, Table 1 briefly summarizes representative examples and the corresponding related applications of these examples.

## 3. Materials for NPTs

Conventionally, PTs are generally made of various rubbers and reinforced with structural components like skeleton materials, such as steel cord and textile fibers, among others [50]. In contrast, the materials selected for NPTs are not limited to rubber but encompass a wide array of elastomers and their composites, and in some instances, even metallic materials are extensively employed. The versatility of NPT design permits the utilization of disparate materials for various tire components, contingent upon specific performance requirements. Consequently, the design flexibility associated with NPTs is notably heightened. Furthermore, the materials utilized in NPTs correspond to their structure and can be categorized into three principal classes: tread materials, support structure materials, and the heterogeneous adhesives that tightly bound the various components together.

**Figure 3:**
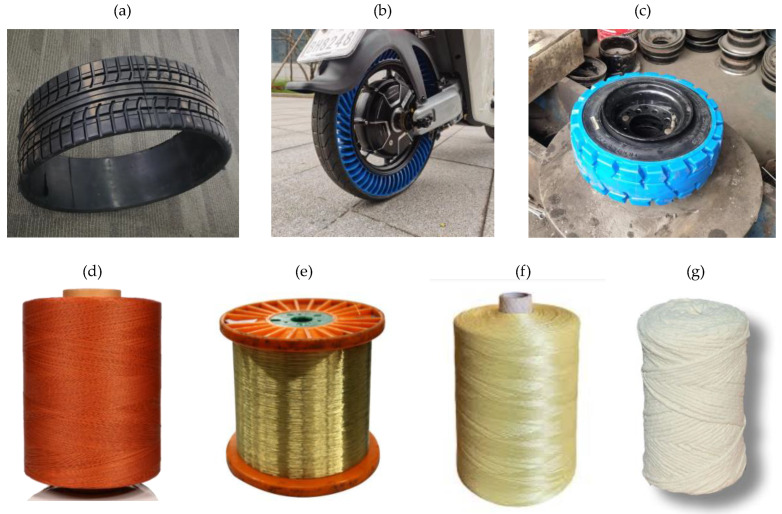
Representative materials used for NPTs: (**a**,**b**) rubber of tread; (**c**) TPU particles; (**d**) nylon cord coated with resorcinol–formaldehyde–latex (RFL) solution; (**e**) steel cord; (**f**) aramid cord; (**g**) twisted rope of glass fibers.

The representative materials are shown in Figure 3.

### 3.1. Tread Combination Materials

#### 3.1.1. Tread Materials

Tire treads necessitate the possession of several vital attributes, including exceptional grip performance; resistance to wear, tear, and punctures; and superior wet skid resistance. In this regard, rubber materials have historically held a dominant position. Correspondingly, akin to PTs, the tread materials of NPTs predominantly comprise rubber compounds. These compounds generally feature rubber as the matrix, augmented by the inclusion of fillers and additives.

Rubber can be categorized into two types: natural rubber (NR) and synthetic rubber. NR comes from nature and represents a renewable resource endowed with excellent mechanical properties after vulcanization. Using natural rubber instead of synthetic rubber is one step toward a greener environment in a variety of technological applications, including the fabrication of vehicle tires [51]. However, due to certain production constraints and limitations associated with specific performance aspects, scientists have endeavored to synthesize rubber to address these deficiencies, including styrene-butadiene rubber (SBR) [52], butadiene rubber (BR) [53], polyisoprene rubber (IR) [54] and others. To enhance the overall performance characteristics, scientists have also resorted to blending multiple rubber types. For example, Pongdhorn et al. [55] investigated the effects of different blend ratios of SBR, BR, and SBR types on the properties of SBR/BR tire tread compounds, revealing that the hardness, strength, and wet grip efficiency were deteriorated whereas the rolling resistance was improved with the increase in BR proportion. Rehman et al. [56] combined IR and BR to produce IR/BR blends and found that increasing the loading of BR had a detrimental effect on the hardness, tensile strength, and elongation at break, albeit it led to improvements in abrasion resistance and thermal stability.

Fillers serve to bolster the matrix’s mechanical properties, with carbon black being the most prevalent filler employed in the tire rubber industry. This choice is predicated upon the material’s exceptional performance attributes and cost-effectiveness. Gao et al. [57] introduced the absolute coordinate system to the digital image correlation method to solve the large deformation of the rubber surface and studied the influence of carbon black on the rolling mechanical properties, therefore providing a new test method for optimizing the formula of tread rubber compounds. Nevertheless, the use of silica in the tire industry is on the rise, primarily driven by the adoption of green tire concepts. Shin et al. [58] synthesized a novel dispersant termed poly (itaconic acideco-acrylamide) (IA-co-AAM) using copolymerized IA and AA and confirmed the improved silica dispersibility in silica–SBR compounds. Consequently, the dynamic viscoelastic properties of the compounds and the rolling resistance of the compounds for the tire tread area were also improved.

Additives represent an essential component within the rubber formulation, encompassing softeners, antioxidants, and vulcanizing agents. These additives play a pivotal role in enhancing the processability of rubber and elevating its mechanical strength. Lovison et al. [52] employed modified soybean oils to replace naphthenic oil in rubber processing and found that the modified soybean oils decreased the glass transition temperature of the rubber compounds, leading to a decrease in the optimal cure time and a simultaneous increase in the crosslink density, ultimately enhancing abrasive wear resistance.

In addition to conventional rubber materials, cast polyurethane is sometimes used as the tread material of solid tires to give full play to its good wear resistance [10,59]. Solid tires with polyurethane tread have good static load performance, excellent wear resistance, good economic performance, and environmental protection significance.

#### 3.1.2. Skeleton Materials

Skeleton materials play a fundamental role in the construction of traditional PTs by serving as the primary stress-bearing layers within rubber products. These materials wield significant influence over tire service performance, longevity, and overall utility. Ideal skeleton materials are characterized by a spectrum of essential mechanical proper-ties, including high strength, requisite modulus, resistance to fatigue, low susceptibility to creep, low density, resilience to extreme temperature fluctuations, corrosion resistance, and flame-retardant attributes. Therefore, the skeleton material is equally important in the manufacturing process of NPTs. A diverse array of materials have the potential to be employed as skeleton materials in NPTs, including steel cord, polyester cord, nylon cord, aramid cord, rayon, glass fibers, and carbon fibers.

Notably, steel cord exhibits critical properties that are indispensable for tire construction. These encompass a high tensile strength, the requisite degree of modulus, and the vital aspect of ductility, all of which are imperative for fulfilling the performance requisites relating to load-bearing capacity, driving responsiveness, and dimensional stability [60,61]. Polyester cord (also called PET fiber) has the advantages of high strength and good elasticity and is widely used as skeleton material in tires. Liu et al. [62] modified PET fibers by introducing phenolic hydroxyl and epoxy ligand groups and filled them into acrylonitrile–butadiene rubber (NBR)/Fe_2_(SO_4_)_3_ coordination composites to further reinforce the performance. Mahdavipour et al. [63] developed a new cord structure using nylon6,6 and polyester yarn and studied the effect of thermal treatment on the thermomechanical properties of the nylon 6,6/polyester hybrid cord. The results show that thermal treatment increased the crystallization of polymers and therefore led to a higher initial modulus and storage modulus in the hybrid cord. Glass fibers have excellent performance and a wide variety of advantages, such as small density, good toughness, high chemical stability, and high temperature resistance. However, glass fibers are burdened with the drawbacks of brittleness and suboptimal wear resistance. Nevertheless, glass fibers can be used as a radial tire buffer layer and bias tire protection layer, and their comprehensive physical properties are the closest to those of steel cord [64]. Sassi et al. preliminarily demonstrated that it is feasible to use glass fiber composites to produce NPTs through static and dynamic testing [65].

### 3.2. Elastic Support Materials

Elastic support materials form the core foundation for endowing NPTs with the necessary support and cushioning performance. These materials are subjected to repetitive stretching and compression during vehicular movement, and the frequency of these mechanical stresses escalates with increasing vehicle speeds. Consequently, elastic support materials are required to exhibit exceptional fatigue resistance.

Metals are suitable for elastic support structures for several reasons: they have high strength and stiffness, a high elastic modulus, a long fatigue life, and good processing performance. Generally, NPTs that use metal as elastic support structures have good load-bearing performance [10,25,66].

In addition, elastomeric materials, due to their mechanical characteristics, are deemed highly suitable for deployment as elastic support materials in NPTs. Among these, PU stands out as a representative elastomer with versatile properties that facilitate the adjustment of various mechanical attributes [67]. Structurally, PU comprises a repeating unit featuring a soft segment and a hard segment. The soft segment is characterized by a lengthy oligomer polyol chain, while the hard segment is composed of diisocyanate and a chain extender. The PU molecular chain contains numerous polar groups that enable the formation of intramolecular and intermolecular hydrogen bonds. Additionally, the microphase separation between the soft and hard segments generates microphase regions. The continuous soft phase imparts elasticity, while the dispersed hard segment reinforces and crosslinks the material [68].

PU can be categorized into two main types based on processing technology: castable polyurethane (CPU) and thermoplastic polyurethane (TPU). CPU is suitable for the process of centrifugal casting, whereas TPU is compatible with injection molding and 3D printing. Valuable research has been conducted in this domain. For example, Michelin prepared Tweel NPT by centrifugal casting using CPU as its spoke material [69]. Ali et al. [22] designed and analyzed various NPTs with different structures of spokes made of TPU. Dezianian et al. [70] employed a multi-material metamaterial, combining TPU with polylactic acid (PLA), to form the spokes of NPTs through a 3D printing method.

While PU demonstrates several remarkable properties, such as high tensile strength, wear resistance, and low rolling resistance when utilized in the production of NPTs’ elastic supports, it still suffers from inherent issues of heat generation under high-load, high-frequency deformation conditions. These issues can lead to problems like melting, tearing, and delamination during the movement of the vehicle, ultimately resulting in a decline in performance, which limits the further use of NPTs. To address these challenges, research has been dedicated to enhancing the heat resistance of PU. For instance, Hu et al. [71] synthesized a PU elastomer made of polycarbonate glycol, 1,5-naphthalene diisocyanate, and 1,4-butanediol with high heat resistance and self-repairable ability and demonstrated that the comprehensive performance of this kind of PU matches that of existing green tires.

TPEs constitute another class of elastomeric materials characterized by attributes such as low density, high chemical resistance, and heat resistance [72]. Broadly, TPEs con-sist of semicrystalline and elastomeric phases, providing them with the processing flexibility inherent to thermoplastics [73,74]. Nevertheless, the use of other TPEs in the production of elastic supports for NPTs, excluding solid-type NPTs, has been scarcely reported. As technology advances, TPEs are poised to find more promising applications within the NPT manufacturing landscape.

In addition to the above materials, the technology of using composite materials 3D printing to form NPT has gradually developed in recent years. Wang et al. [75] proposed a carbon fiber-reinforced polyethylene terephthalate (PET/CF) honeycomb as a support structure for NPTs, which can be easily prepared using 3D printing technology. The PET/CF honeycomb as the support structure of the NPT showed outstanding bearing capacity and stiffness in contrast with its elastomer counterparts.

### 3.3. Heterogeneous Bonding Adhesives

NPTs face internal bonding challenges due to the heterogeneous nature of the materials involved, particularly at the interfaces between the tread and the skeleton materials, the tread and the elastic support structure, and the elastic support structure and the metal rim. To ensure effective bonding between various components of an NPT and to enhance its overall stability and integrity, it is crucial to use advanced heterogeneous surface adhesives with high bonding strength. A summary of the bonding conditions among these materials is provided below.

1. The adhesion of skeleton materials with the tread:

The tread material in NPTs is mostly rubber, so the bonding between the skeleton materials and the tread is essentially the bonding between the skeleton materials and rubber. The strength of this bond is critical to tire performance [76], as pneumatic tire issues like bursts, fatigue, delamination, and other damages often result from adhesion failures between rubber and skeleton materials. Strong adhesion directly influences the tire’s performance and lifespan.

The adhesion between rubber and skeleton materials, whether steel wire, polyester, nylon, aramid, or glass fiber composites, has been extensively studied. Chowdhury et al. [77] investigated the effect of adhesive ingredients on the adhesion between rubber and brass-plated steel cords, finding that cobalt stearate improved adhesion strength by 12% (without aging) and 11% (after humid aging) compared to a control compound. Furthermore, Du et al. [78] developed a mussel-inspired bonding method to improve interfacial adhesion between nylon cords and rubber composites by coating silica with polydopamine. These approaches enhanced the durability and performance of NPTs by improving adhesion at critical interfaces. Epoxy-based adhesives, widely used for bonding metal to rubber and other polymeric materials in NPTs, are favored for their excellent mechanical properties and chemical resistance. Recent advancements have focused on incorporating nano-fillers like silica and alumina to improve toughness and adhesion strength when used with epoxy adhesives [79].

The RFL system (resorcinol–formaldehyde–latex system) is the most widely used chemical bonding system between skeleton materials and rubber. Its application in tires can be traced back to the 1940s [80]. The RFL system forms a strong bonding interface between the skeleton materials and the rubber matrix through chemical crosslinking and physical adhesion, thereby enhancing the mechanical properties and durability of composite materials [81]. Common skeleton materials used with the RFL system include steel cords, polyester (PE), nylon, aramid, and rayon [82]. Inorganic fiber skeleton materials, such as basalt fiber, can also be treated with the RFL system [83]. In addition, the use of glass fiber as a skeleton material in airless tires is on the rise due to its high tensile strength, corrosion resistance, and lightweight properties. Glass fiber is mainly used in two forms, both of which can utilize the RFL system to improve the adhesion between glass fiber and rubber. First, similar to traditional cord materials like nylon or polyester, glass fiber can be woven into cords or ropes. In this case, to enhance the adhesion between glass fiber and the RFL system, silane coupling agents are usually applied as a pre-treatment, followed by the application of the RFL adhesive system onto the surface of the treated glass fiber cords or ropes. Secondly, glass fibers can be combined with polymeric materials, such as epoxy resin, to form glass fiber-reinforced composites [84]. Epoxy resin, as a matrix material, typically exhibits high strength and chemical stability. Therefore, before applying the RFL system, the surface of the epoxy resin must undergo appropriate treatment to improve its adhesion with the RFL system. Common surface treatment methods include sanding and cleaning, chemical modification, and plasma treatments.

2. The adhesion of the elastic support structure with the tread:

Depending on the design requirements, materials used for the elastic support structure can include metals, rubber composites, PU, TPE, and others, and tread materials may also include rubber and PU. Consequently, the bonding between the elastic support structure and the tread presents a series of complex heterogeneous surface bonding challenges, such as metal-to-rubber, metal-to-PU, PU-to-rubber, and rubber-to-rubber bonding. Corresponding adhesives are required to address the complex stress fields, such as shear, tensile, and compressive stresses, that may occur at various bonding interfaces under different operating conditions of NPT, as well as the issue of high-temperature fatigue under complex deformation and heat generation conditions.

Currently, the most widely used materials for the elastic support structure are rubber and polyurethane, while rubber is the most common material for tread. In most cases, the types of rubber used for the tread and the elastic support structure differ. Therefore, the most common heterogeneous surface bonding types between the elastic support structure and the tread involve rubber-to-rubber and polyurethane-to-rubber adhesion.

One common method for bonding rubber to rubber is thermal vulcanization, a process in which two pieces of vulcanized rubber are bonded together through heat and pressure. In this method, the adhesive used is raw rubber, typically cold-flip adhesive buffer glue. During vulcanization, the unvulcanized rubber forms new crosslinks with the raw rubber to be bonded, creating a strong adhesion.

Another common method for bonding rubber to rubber is the use of specialized rubber adhesives, with polyurethane adhesives being the most widely used. Polyurethane adhesives are renowned for their flexibility and excellent bonding capabilities, particularly in heterogeneous surface bonding. They can bond polar groups such as amino esters, isocyanates, and ureas, which form covalent and hydrogen bonds with materials containing active hydrogen. This makes them suitable for bonding various materials, including metals, rubber, plastics, wood, leather, fabric, paper, and ceramics, by simply adjusting the formulation [85]. Polyurethane adhesives have several key properties that make them ideal for heterogeneous bonding in NPT applications: (1) versatility—PU adhesives can be formulated to meet the specific bonding requirements of various substrates, making them suitable for NPT manufacturing processes; (2) strong adhesion—they can form strong bonds with a wide range of materials, making them especially suitable for applications requiring high tensile and shear strengths; (3) chemical resistance—PU adhesives exhibit strong resistance to chemicals, moisture, and temperature variations, which is crucial in the harsh environments where NPTs are used.

When using polyurethane adhesives to bond vulcanized rubber, the rubber usually requires surface treatment. This is because common rubbers used in tires, such as natural rubber and butadiene rubber, are low-polarity materials that have poor affinity with polyurethane. In such cases, improving the polarity of the rubber surface through physical or chemical methods is essential for ensuring strong adhesion. The most common chemical method is oxidation. For example, Verónica et al. [86] demonstrated that the chemical bonding formed between trichloroisocyanuric acid (TCI) solution and the rubber surface enhances adhesion strength when used with polyurethane adhesives. Kapica et al. [87] increased peel strength from 2.6 kN/m to 18.5 kN/m by treating SBS rubber with a plasma surface treatment before applying polyurethane adhesives.

Other types of heterogeneous surface bonding between the elastic support structure and the tread, such as rubber-to-metal and polyurethane-to-metal bonding, can also utilize polyurethane adhesives. For example, Liu et al. [10] designed a high-load-capacity NPT, where heterogeneous bonding between the elastic support structure and the tread involved bonding metal to polyurethane. Deng et al. [24] explored similar bonding between metal and rubber in the elastic support structure and the tread. Like rubber, metal surfaces can be treated through a plasma surface treatment or chemical modification to alter their surface polarity and improve bonding with materials such as polyurethane. For instance, Ochoa-Putman et al. [88] conducted a series of studies on metal surface modifications that enhanced their compatibility with polyurethane bonding.

Bonding polyurethane to vulcanized rubber presents a technical challenge because these two materials have different chemical characteristics and physical properties. There are two primary methods for bonding polyurethane to vulcanized rubber: bonding after PU curing and curing PU directly onto rubber. Teppa et al. [89] studied both bonding methods and found that curing PU directly onto rubber resulted in bonding strength 70 times higher than that achieved with bonding PU after curing. It is important to note that surface treatment is an essential step to improve the bonding strength between rubber and polyurethane. The purpose of this step is to increase surface roughness, clean contaminants, and enhance the surface’s chemical activity. Common surface treatment methods include mechanical sanding, chemical treatment, plasma treatment, or corona treatment. After surface treatment, adhesive primers must be applied, with common adhesives including polyurethane adhesives, acrylic adhesives, and epoxy adhesives.

3. The adhesion of the elastic support structure with the rim:

The rim is typically made of metal, so bonding issues between the elastic support structure and the rim generally involve metal-to-metal, rubber-to-metal, and PU-to-meta connections. The metal elastic support structure and metal rim are typically connected through mechanical means [23], while the rubber or polyurethane elastic support structure requires strong heterogeneous surface adhesives to bond with the metal hub. The relevant adhesives and surface treatment methods have been discussed earlier, so they will not be elaborated on here.

## 4. Processing Methods for NPTs

**Figure 4:**
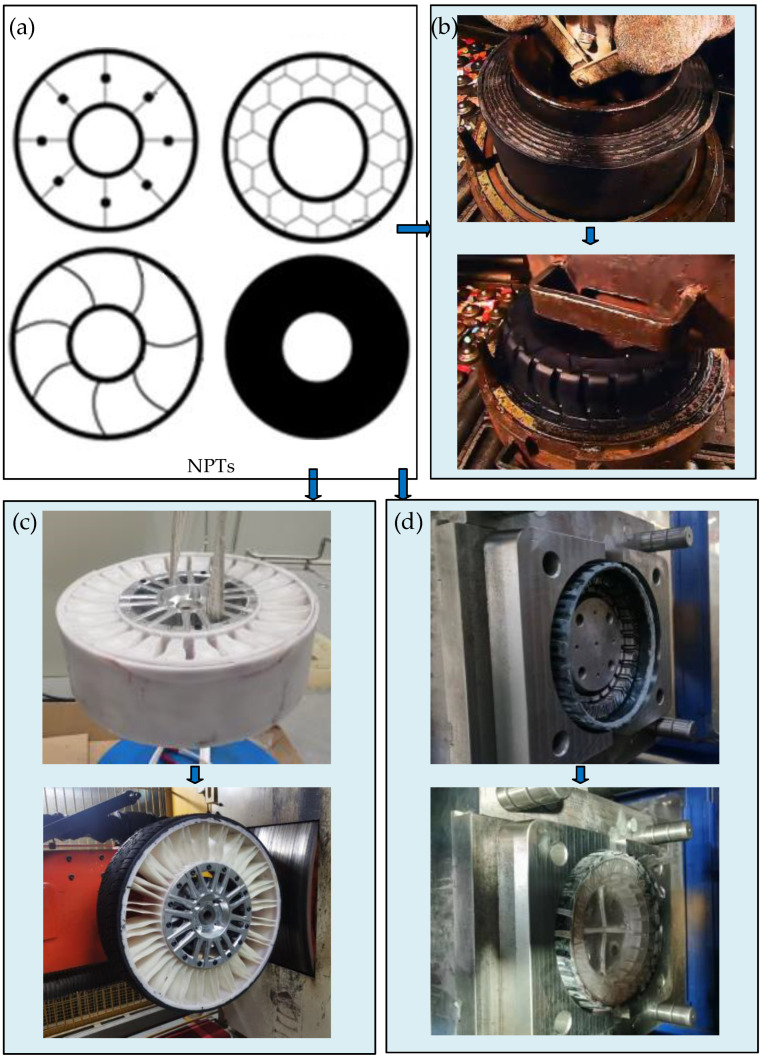
Main processing methods: (**a**) representative NPTs; (**b**) one-piece molding; (**c**) from the interior to the exterior; (**d**) from the exterior to the interior.

Significant differences exist between NPTs and PTs, primarily manifesting in their structural attributes, notably the intricate elastic support system within NPTs. In addition, the manufacture of NPTs enjoys a broader array of material selection options. Consequently, conventional tire production techniques are not directly transferrable to NPT manufacturing due to variances in both structural design and the material used. The non-pneumatic tire-molding process can be mainly divided into three types: from the interior to the exterior, from the exterior to the interior, and one-piece molding, as shown in Figure 4. The process starting from the interior to the exterior means that the internal elastic support structure is molded first, and then the external tread is molded on this basis. The process starting from the exterior to the interior is the opposite, first molding the external tread and then molding the internal elastic support structure. The last process, one-piece molding, involves the external tread and the internal elastic support structure being processed and molded at the same time.

**Figure 5:**
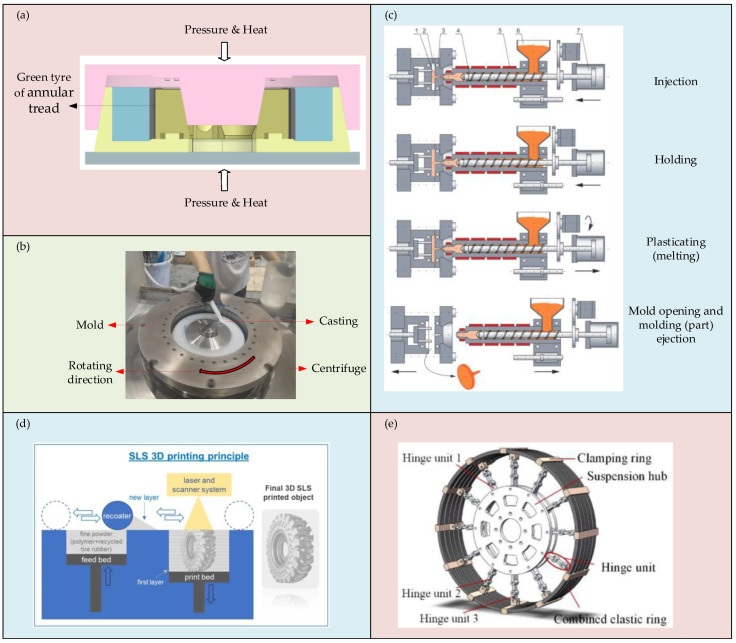
Main processing methods: (**a**) a schematic diagram of compression molding; (**b**) the centrifugal casting process; (**c**) a schematic diagram of the injection molding process [90]; (**d**) a schematic presentation of the SLS 3D printing process [91]; (**e**) a schematic diagram of mechanical assembly [92].

At present, regardless of the above molding process, the processing methods of NPTs encompass compression molding, casting, centrifugal casting, injection molding, 3D printing, and mechanical assembly, as illustrated in Figure 5.

Table 2 provides a comprehensive summary of the materials and structural configurations of NPTs suitable for various molding methods. Additionally, it includes illustrative product images for reference.

### 4.1. Compression Molding

Compression molding is a manufacturing method that is prevalent in the production of PTs, but its utilization in NPT fabrication is relatively less common. It is primarily applied in the production of solid-type NPTs, annular/strip pre-cured tread, or NPTs with simple elastic support structures. The compression molding process commences with the meticulous selection of suitable materials for the tire, which typically involves the utilization of thermoplastic or thermosetting rubber compounds, along with other elastomeric materials. These materials are chosen due to their inherent attributes of durability, resilience, and resistance to wear and abrasion. Subsequently, a mold is meticulously designed to assume the precise shape of the intended NPT. This mold is custom-configured to impart the desired tire dimensions, tread pattern, and other pertinent features. It is usually fabricated from high-strength steel or other robust materials capable of withstanding the pressure and elevated temperature inherent to the molding procedure. The compression process unfolds with the introduction of preheated material into the mold cavity, followed by the application of pressure to compress and sculpt the material into the designated tire structure. After the compression phase, the material undergoes a controlled cooling and solidification process, where parameters such as cooling time and temperature are meticulously regulated to ensure the ultimate structural integrity and performance attributes of the tire.

The vulcanization and curing process during compression molding are important processes in the fabrication of solid-type NPTs. Given the substantial thickness of solid-type NPTs, the curing process typically unfolds under transient non-isothermal conditions, where the temperature distribution within the mold significantly influences the spatial distribution of curing levels throughout the entire product. Limrungruengrat et al. [95] utilized the commercial finite element software ABAQUS (v. 2016, ABAQUS Documentation, Dassault Systèmes, Providence, RI, USA) to conduct numerical simulations of the curing process of a solid-type NPT comprising three layers of different rubber compounds and evaluate the results of cure level distribution within the tire. The simulation outcomes underscored that the heating duration of solid-type NPTs can be significantly reduced when the mold temperature and initial temperature of rubber are increased.

### 4.2. Casting

Casting represents a well-established manufacturing process commonly applied for crafting components and products constructed from an array of materials, including materials, plastics, ceramics, and more. The primary purpose of this process is to attain the desired shape and dimensions through the introduction of molten material into a mold, enabling subsequent cooling and solidification [96]. Sometimes, in order to improve the molding accuracy of the product, make the hardness more uniform, and improve production efficiency, the mold can be rotated during the pouring process, which is known as centrifugal casting. Compared to compression molding, the process of centrifugal casting is notably simplified due to the utilization of materials with low viscosity and high fluidity.

Centrifugal casting typically consists of five steps in accordance with established technical protocols. Initially, the design and construction of a mold matching the intended configuration of NPTs is imperative. This mold may either consist of a single unit or be composed of multiple components, particularly when addressing intricate elastic support structures. Subsequently, the synthetic raw materials of PU are heated and blended using specific equipment. The resulting mixture is then introduced into a rotating mold, enabling the material to effectively infiltrate all intricacies of the mold and facilitating the removal of any air bubbles within the mixture. During the subsequent phase, chemical reactions within the mold transpire over a defined period to endow the NPT with the requisite mechanical properties. Following this, the mold undergoes a cooling process. The culminating step entails the extraction of the solidified product from the mold, often requiring additional measures such as material trimming or surface finishing to achieve the requisite NPT quality.

NPTs predominantly fabricated from PU can be effectively produced through the centrifugal casting method [10,97]. Bert et al. [98] studied the manufacturing of a Michelin Tweel NPT to investigate the influence of the entire life cycle of NPTs on the environment. The production of Tweel NPT involved a tripartite procedure encompassing the processing of the tread, hub, and PU spokes. The centrifugal casting method was employed specifically for crafting the PU spokes. In this process, the hub and the tread are concentrically situated within the mold and PU is poured into a spoke and shear band mold while the entire assembly is rotating. This rotation ensures the comprehensive filling of the mold in the radial direction. Subsequent to the pouring procedure, the entire Tweel assembly ceases its rotation and is subsequently transferred to a secondary curing chamber. This definitive curing process is conducted at a temperature of 100 °C for a duration of 4 h, resulting in the attainment of the targeted PU attributes and the establishment of robust component adhesion within the Tweel NPT.

### 4.3. Injection Molding

Injection molding is a pivotal and sophisticated processing method that transforms thermoplastic materials into diverse products of varying shapes and configurations. This method is widely used owing to its exceptional precision in size, rapid production rates, repeatability, and capacity to fabricate the mass production of intricately designed components [99]. Technically, injection molding operates as a cyclic process, which unfolds through distinct stages encompassing filling, packing–holding, and cooling [100]. This method involves the elevation of thermoplastic materials to high temperatures, transforming them into a molten state, and subsequently injecting them into a mold with the aid of specialized machinery. It is imperative that this process transpires expeditiously and at precise temperatures to forestall incomplete material filling. Following the complete infusion of the mold, the cooling phase ensues, the duration of which varies contingent upon the type of materials employed and the thickness of the product. Additional steps such as trimming, painting, or assembling parts may be needed for the final product [101]. With the development of technology, many more advanced injection molding technologies have evolved from the foundational principles of injection molding, encompassing water-assisted injection molding [102], gas-assisted injection molding [103], microcellular injection molding [104], variable mold temperature technologies [105], microinjection molding [106], and rapid thermal cycling molding [107].

Within the domain of NPT manufacturing, injection molding stands as a pivotal molding method. Zhu et al. [108] employed a finite element analysis (FEA) software called Moldflow (https://www.autodesk.com/products/moldflow-design/subscribe, accessed on 13 November 2024) to analyze NPTs and revealed that the parameters of the injection molding process, including the injection speed, injection pressure, packing pressure, feed temperature, mold temperature, and others, can be accurately predicted through this technique. Therefore, the repetitive process of mode adjustments and testing is rendered unnecessary. This not only enhances product quality and production efficiency but also reduces cost overheads. He et al. [93] advanced the notion that the rational design of injection molds for NPTs exerts a direct influence on processing quality and tire service life. It was found that the semi-annular follow-up cooling channel arrangement significantly reduces the pressure loss within the cooling loop, diminishes the cycle time and cooling time by 77% and 9.6%, respectively, and reduces tire volume shrinkage by 0.012%. Consequently, this design optimization enhances cooling uniformity, mitigates energy losses, and augments the quality of NPTs produced via injection molding.

### 4.4. Three-DimensionalPrinting

The emergence of 3D printing technology has been heralded as a pivotal component of the fourth industrial revolution, affording the potential for substantial resource, cost, and time savings in the production phase [109]. In addition, 3D printing technology, also known as additive manufacturing (AM) technology, can bring new advantages to industries by decentralizing production, driving product customization, reducing production complexity, improving time to market, improving resource efficiency, and rationalizing inventory and logistics [110]. This technology empowers the creation of products that are both lighter and stronger, all while obviating the necessity for assembly and molds during the manufacturing process [111]. Therefore, 3D printing technology is an ideal processing method for the production of NPTs.

In 3D printing, a designed 3D object is manufactured layer by layer through a computer-controlled translation phase based on data from a 3D computer model. Depending on the specific printing process and materials employed, the print head or laser optics are used to deposit or fabricate one layer of 3D objects at a time [112]. Consequently, the initial phases of modeling and performance simulation are particularly important for NPT products with intricate structures. Narasimhulu et al. [113] comparatively analyzed three distinct cellular structures to develop simplified NPT models and employed FEA-based software to assess the static loading conditions associated with a standard passenger vehicle. Thereafter, CAD models were generated, and a static performance analysis was conducted in ANSYS software (https://www.ansys.com/, accessed on 13 November 2024). This study concluded that the reentrant cellular-type NPTs are the most suitable ones for city vehicles. Jafferson et al. [114] used NTopology to design NPTs with various structures and compared the weight and materials’ cost when using TPU materials using BigRep. Alireza et al. [115] explored the mechanical properties of NPTs manufactured by fused deposition molding (FDM) 3D printing technology and investigated the radial response of NPTs with different unit cell numbers, thicknesses, and outer diameters. Volochko et al. [116] experimentally investigated the influence of filling density of parts manufactured by 3D printing on their mechanical properties and the application of the obtained results for the production of airless tires by using FDM technology.

To facilitate this endeavor, a robust finite-strain beam element was developed to simulate the proposed cellular NPTs, which is a method formulated based on the nonlinear Green–Lagrange strain tensor and Mooney–Rivlin hyperelastic constitutive model.

### 4.5. Mechanical Assembly

In terms of structure characteristics, mechanical elastic support-type NPTs exhibit a similarity to spoke-type NPTs. However, the distinguishing feature of the former lies in its primary composition, which predominantly comprises metallic materials, necessitating the fabrication of this type of NPTs through mechanical assembly. The research team led by Zhao Youqun at the Nanjing University of Aeronautics and Astronautics has made notable contributions in this field. The structure of mechanical elastic support-type NPTs primarily encompasses a suspension hub, hinge units, combined elastic rings, and a flexible tire body [117]. Each of these components is independently prepared in accordance with the designated design specifications and is subsequently assembled, and the rubber tread adheres to the outermost surface of the tire as the final step. The specific steps are as follows: initially, the elastic ring is wound with steel wire and uniformly fixed by the snap ring distributed along the circumference of the tire; subsequently, the combination of snap rings, elastic rings, and a flexible tire body undergoes vulcanization to yield a unified structure; lastly, hinge units are employed to establish the connection between the suspension hub and the elastic outer ring [118].

## 5. Conclusions and Prospects

The manufacture of NPTs is systematic engineering process. In the initial stages, structural designers first create a preliminary model of NPTs and input material parameters based on their own experience into simulation software. This is carried out to obtain the performance parameters of the preliminary tire model, such as stiffness, rolling resistance, and tread contact patterns. Based on these tire performance parameters, adjustments are made to the tire model and more suitable performance parameters are chosen, continuously optimizing the tire’s structural design. This iterative process leads to the final structural model of NPTs and the selection of materials with specific performance characteristics. Soon afterwards, process engineers select appropriate processing methods based on the final structural model of NPTs and design suitable molds. During this process, process engineers provide feedback to the structural designers from a process perspective, considering factors like process conditions and the feasibility of tooling. Meanwhile, materials researchers, guided by the tire’s performance requirements, select commercially available materials with suitable properties or synthesize new materials and make modifications to meet the requirements set by the structural designers as closely as possible. Throughout this entire process, engineers responsible for three aspects—structure, process, and materials—are in constant communication, providing feedback to one another and making iterative improvements. This collaborative effort ultimately leads to the manufacture of NPTs with the desired performance.

NPTs have obvious advantages in specific application scenarios, but there are also some disadvantages in many designs, including poor comfort, large weight, limited elasticity, high manufacturing complexity, and high cost. Therefore, it is believed that the future development direction may involve the following aspects:

1. Material innovation and processing method improvement: The development of NPTs in the future will focus on material innovation and processing method improvement. Researchers will develop more wear-resistant, tear-resistant tread materials, fatigue-resistant elastic support materials, high-temperature dynamic bonding adhesives, and compression-resistant, fatigue-enduring skeleton materials in order to improve the durability and performance of tires. In the meantime, manufacturers will also seek to improve processing methods to promote the large-scale production of NPTs.
2. Sustainability and environmental protection: The manufacture and use of NPTs may evolve in a more sustainable and environmentally friendly direction. The use of renewable materials, reducing the energy consumption and emissions of the production process, and improving the recycling efficiency of tires are likely to be the development trends of the future.
3. The extensive application of additive manufacturing technology: Additive manufacturing technologies, such as 3D printing, are expected to play a greater role in the future processing of non-pneumatic tires, particularly in areas like customization, rapid prototyping, and small-batch production. These technologies enable the precise molding of tire structures and the easy customization of complex geometries, thereby accelerating the design, prototyping, and testing processes. Moreover, 3D printing reduces mold costs, leading to a shorter and more cost-effective development cycle for innovative tire designs.
4. Intelligent production and automated control: To enhance production efficiency and reduce costs, the manufacturing processes for non-pneumatic tires will increasingly trend toward smart and automated systems. By utilizing intelligent manufacturing systems and sensing technologies, real-time monitoring and quality control of production processes can be achieved, improving efficiency and product consistency. In the future, machine learning algorithms may be integrated to optimize production parameters and processes, enabling automatic adjustments and improvements in manufacturing workflows, thereby further enhancing precision and efficiency in production.
5. Comfort and shock absorption improvement: Manufacturers may pay more attention to ride comfort and shock absorption for future NPTs. Through more optimized material and structural design, vibration and noise can be reduced, providing a smoother driving experience.
6. Intelligence and connectivity: With the intelligent development of the automotive industry, it is also possible for NPTs to relate to the intelligent system of the vehicle to monitor tire status, air pressure, and temperature in real time and provide relevant data and alerts. NPTs may perform better in different road conditions because they are not affected by changes in tire pressure. Future developments are likely to emphasize the versatility of tires, adapting to a variety of roads and environments.
7. Customized design: Manufacturers may pay more attention to personalized and customized design in future NPTs to meet the needs and preferences of different users.

In short, the future development direction of NPTs will cover many fields such as materials, processing methods, performance, environmental protection, and intelligence. The synthesis of new high-performance materials is the core element that enables the further development of NPTs. These trends will help drive the use of non-pneumatic tires in the automotive industry, improving their performance and sustainability.

## Figures and Tables

**Table 1:** Categories of NPTs.

Application			Representative Illustration	Reference
Mechanical elastic support type		Areas with medium/low speed and medium/high load	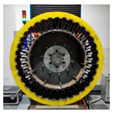	[10]
			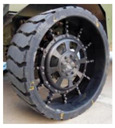	[11,23,24]
Elastomer elastic support type	Spoke type	Areas with medium speed and load/areas with high speed and light load	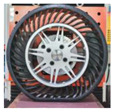	[19,20,43,44,45,46]
	Honeycomb type		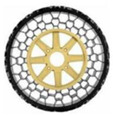	[11,47,48]
	Cellular type		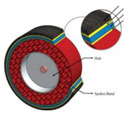	[32,49]
	Cross-supported type		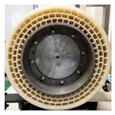	[36]
	…		…	…
Solid type		Areas with low speed but high load	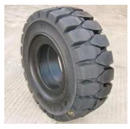	[39,40,41]

**Table 2:** Comprehensive summary of the materials and structural configurations of NPTs.

Processing Method	Application	Representative Illustration	Available Materials	Applicable Molding Process for NPTS	Reference
Compression molding	Solid-type NPTs, annular/strip pre-cured tread, NPTs with simple elastic support structures	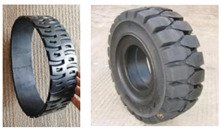	Rubber/PU	From the interior to the exterior/from the exterior to the interior/one-piece molding	[39]
Casting	Support structures, tread	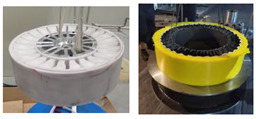	CPU	From the interior to the exterior/from the exterior to the interior	[10]
Injection molding	Support structures	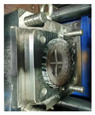	TPU	From the interior to the exterior/from the exterior to the interior	[93]
3D printing	Support structures, entire NPT	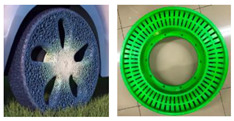	TPU/FET/Rubber	From the interior to the exterior/from the exterior to the interior/one-piece molding	[14,94]
Mechanical assembly	Support structures	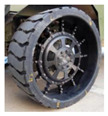	Metal	From the interior to the exterior/from the exterior to the interior	[11,92]
